# Validation of an online course on introduction to entrepreneurship and innovation in nursing and health

**DOI:** 10.1590/0034-7167-2024-0614

**Published:** 2025-12-08

**Authors:** Jouhanna do Carmo Menegaz, Aurilívia Carolinne Lima Barros, Letícia de Lima Trindade, Thais de Fátima Aleixo Corrêa, Matheus Moraes Silva, Emily Silva Pontes

**Affiliations:** I Universidade do Estado de Santa Catarina. Chapecó, Santa Catarina, Brazil; II Barros Tecnologia LTDA. Caxias, Maranhão, Brazil; III Faculdade Estácio do Pará. Belém, Pará, Brazil; IV Universidade Federal do Pará. Belém, Pará, Brazil; V Fundação Universitária Evangélica. Anápolis, Goiás, Brazil

**Keywords:** Entrepreneurship, Nursing, Validation Study, Health, Health Personnel., Emprendimiento, Enfermería, Estudio de Validación, Salud, Personal de Salud.

## Abstract

**Objectives:**

to validate an online course on introduction to entrepreneurship and innovation in nursing and health.

**Methods:**

this methodological study focused on the structural and content validation of the *Introduction to Entrepreneurship and Innovation in Nursing and Health Course*. Ten expert judges participated in the validation process and completed a questionnaire to assess the course’s validity. The validation used the following parameters: Content Validity Index, Content Validity Coefficient, and exact binomial test.

**Results:**

the course was validated in the first evaluation round. The expert judges highlighted the lack of faculty and professionals with well-developed entrepreneurial skills, the curriculum’s emphasis on care-based training, and logistical issues related to the course.

**Final Considerations:**

the findings demonstrate high structural and content validity of the course, highlighting its theoretical and methodological quality and its alignment with the current context, which demands greater access to programs that foster entrepreneurial training in nursing and health.

## RESUMEN

**Objetivos:**

validar un curso virtual de introducción al emprendimiento e innovación en enfermería y salud.

**Métodos:**

estudio metodológico centrado en la validación estructural y de contenido del *Curso de Introducción al Emprendimiento e Innovación en Enfermería y Salud*. Diez jueces expertos participaron en el proceso y completaron un cuestionario para evaluar la validez del curso. Se usaron: Índice de Validez de Contenido, Coeficiente de Validez de Contenido y la prueba exacta binomial.

**Resultados:**

el curso fue validado en la primera ronda de evaluación. Los jueces señalaron la escasez de docentes y profesionales con competencias emprendedoras desarrolladas, la preponderancia del enfoque asistencial en el currículo y cuestiones logísticas del curso.

**Consideraciones Finales:**

los hallazgos muestran una alta validez estructural y de contenido del curso, resaltando su calidad teórica y su pertinencia en el contexto actual, que exige mayor acceso a programas que fomenten la formación emprendedora en enfermería y salud.

## INTRODUCTION

The concept of entrepreneurship encompasses a wide range of definitions, from the sole pursuit of profit to those related to entrepreneurial behavior^(1)^. Regardless of the definition, the entrepreneur engages in a dynamic and creative process of identifying and seizing opportunities. In this context, they stand out by adopting a forward-looking approach oriented toward innovation, continuous planning, and calculated risk-taking to generate financial, scientific, and/or technological value^(2)^.

In nursing, entrepreneurship expands the scope of professional practice, opens up career opportunities beyond hospitals and other public or private healthcare facilities, enhances professional visibility through science and innovation, and addresses gaps in the current healthcare system related to the development of products, training, and educational or research services^(3,4)^.

Nurses apply their technical and scientific knowledge with a focus on evidence-based practice and ongoing professional development; at the same time, they are capable of developing and managing ideas, businesses, and sustainable, efficient technological products^(5)^. On one hand, nursing is grounded in the technical and scientific foundations of caregiving practices. On the other, the profession has increasingly consolidated an entrepreneurial profile that, while still far from reaching its full potential, has already charted its path across various professional domains in Brazil.

Given the range of opportunities that entrepreneurship offers nurses, it must be fostered and its challenges must be addressed. Many nurses still struggle to integrate it into their professional practice. Some individuals have difficulty understanding how entrepreneurial competencies can help them enhance their professional performance and improve both quality of care and client experience. Others, particularly entrepreneurial nurses, face barriers such as insufficient knowledge of bureaucratic procedures, business and financial management, pricing strategies, and client acquisition, in addition to the limited societal recognition of nurses as entrepreneurs^(6)^.

To address these barriers, entrepreneurial education is essential, as it equips professionals with specific knowledge across various areas of weakness within nursing practice. Since entrepreneurship involves competencies not covered by the curricula of most nursing programs in Brazil, these gaps must be filled so that nurses can enter and establish a foothold in the world of entrepreneurship^(7)^.

Fostering entrepreneurial education and promoting innovation are crucial for transforming professional mindsets in nursing through science. To achieve this, nursing curricula must incorporate frameworks that support innovation in education, practice, and research, as well as promote partnerships with other professionals and disciplines that encourage problem-solving^(8)^.

In addition to integrating these topics into academic curricula, other initiatives can help introduce core concepts of entrepreneurship into professional practice. Extracurricular courses help nurses develop key competencies such as business planning, financial organization, and management-skills that are essential for launching their own ventures^(9)^.

There is strong evidence that introducing entrepreneurial education during undergraduate training can yield meaningful benefits for the healthcare sector as a whole. Its inclusion in continuing education supports the development of clear and applicable entrepreneurial competencies. This, in turn, fosters distinctive characteristics essential to advancing nursing practice, such as proactivity, critical thinking, a drive to innovate and plan, self-confidence, commitment to evidence-based action, negotiation and risk management skills, leadership, and interpersonal collaboration, among others^(10)^.

The scarcity of entrepreneurial education courses specifically designed for nursing-and focused on overcoming knowledge-related barriers in this highly relevant and timely field-prompted the development of the *Introduction to Entrepreneurship and Innovation in Nursing and Health Course*. This course aims to support the development of entrepreneurial competencies among nursing professionals. Its availability is intended to bridge the knowledge gap on entrepreneurship in nursing and health by providing a conceptual foundation along with reflective and practical activities.

To ensure the quality of the course’s structure and content, a validation process was proposed involving expert nurses to offer an updated and validated version to nursing and healthcare professionals in Brazil and beyond.

## OBJECTIVES

To validate an introductory course on entrepreneurship and innovation in nursing and health.

## METHODS

### Ethical aspects

This study followed national (Resolution No. 466/2012) and international ethical guidelines. The Research Ethics Committee for Human Subjects of Santa Catarina State University (UDESC) approved it under Final Opinion No. 5.440.353. All participants signed the Informed Consent Form (ICF).

### Study design, period, and setting

This methodological study aimed at validating the structure and content of the *Introduction to Entrepreneurship and Innovation in Nursing and Health Course*. The study was conducted online using a digital research form. The course was designed to train nursing students and professionals and was carried out between 2022 and 2023. Its development followed the Analysis, Design, Development, Implementation, and Evaluation (ADDIE) model, a structured framework commonly used to create educational programs. The course was delivered remotely via a Massive Open Online Course (MOOC) platform provided by Santa Catarina State University (UDESC). Its validation process followed all five phases of the ADDIE model: Analysis, Design, Development, Implementation, and Evaluation.

The research protocol was registered on the Open Science Framework platform (OSF.io) under DOI https://doi.org/10.17605/OSF.IO/PCSYB. The SQUIRE checklist^(11)^ was used to guide the development of the research report.

### Sample, inclusion and exclusion criteria

Fifteen expert judges were invited to participate in the course validation. All were nursing professionals with experience in management, clinical practice, teaching, and relevant scientific output, selected based on criteria outlined in an instrument adapted from Fehring’s scoring system^(12)^. To be eligible, participants had to be nursing professionals and reach the minimum cutoff point of 5 on the selection instrument.

Of the 15 invited judges, five did not respond to the invitation and were therefore excluded from the study. All those who agreed to participate surpassed the cutoff point established by Fehring’s criteria^(12)^, thus qualifying the remaining ten judges to participate in the validation process.

The selection instrument consisted of seven evaluative items. These items addressed academic qualifications, research activity in the field, scholarly output, pedagogical training in health or related fields, and professional experience of more than two years. The maximum possible score was 12 points.

Regarding compliance with the selection criteria, 1 judge scored 11 points, 6 judges scored 9, 1 scored 8, and 2 judges scored 5. The criterion “At least two years of professional experience in the field” was met by all ten judges, indicating solid expertise in their respective areas of practice. In addition, the criteria “Publication of an article, book, or book chapter” (met by eight judges) and “Teaching experience in health, IT, nursing, or related fields” (met by seven judges) show that these professionals also had significant academic productivity and teaching experience. These findings suggest that the judges possessed a robust practical and academic foundation to support their assessments.

### Study protocol

The judges completed a questionnaire to assess the course’s validity, which was structured according to the instructional model adopted-the ADDIE model. The questionnaire was made available in electronic format and distributed via the WhatsApp messaging application. Based on the judges’ responses, the structural and content validity of the course was evaluated, as outlined in the study objective.

The course was developed following the phases of the ADDIE model, and the evaluation process mirrored this framework. The model comprises five phases: Analysis, Design, Development, Implementation, and Evaluation.

- **Analysis** involves mapping the nature of the problem to be addressed, the context in which it is embedded, and the target performance outcomes;
- **Design** includes creating a detailed lesson plan that outlines the content, support materials, course structure, prerequisites for participation, and the proper sequencing of content to promote learning;
- **Development** refers to the creation of the course content, definition of teaching strategies, selection of didactic and technological tools and resources, development of exercises and learning activities, as well as the interaction formats and evaluation methods;
- **Implementation** consists of putting the course into practice;
- **Evaluation** involves assessing learners’ performance to measure the effectiveness of the course, using both formative and summative assessments.

These phases were evaluated using a validation instrument composed of seven sections. The first two sections corresponded to the ICF and general instructions. The remaining five sections addressed the course evaluation based on the ADDIE model, which guided both course development and the validation structure. These criteria served as guiding parameters from the initial development through the evaluation phase, ensuring consistency between the instructional design and the validation framework.

Each thematic section of the evaluation instrument included multiple-choice questions formatted on a four-point Likert scale, followed by an optional open-ended question to collect suggestions, recommendations, or justifications related to the previous answers. The Likert scale consisted of the following options: Disagree, Partially agree, and Totally agree. Each block of questions corresponded to a phase of the ADDIE model.

In the Analysis phase, the problems, the context in which they occurred, the target performance, the types of educational processes to be used, the definition of the target audience, and the critical issues requiring educational actions were identified.

In the Design phase, the development of a detailed lesson plan, the content, support materials, and course structure were assessed.

During the Development phase, when the course content was created, judges evaluated the teaching strategies, tools, didactic and technological resources, learning activities and exercises, interaction strategies, and evaluation methods. Faculty were also assigned to specific course activities during this phase.

The Implementation phase included onboarding and guidance activities, student orientation, and course administration.

Finally, in the Evaluation phase, learners’ performance was assessed to determine the course’s effectiveness. The evaluation was carried out using multiple methods, incorporating both formative and summative assessments.

### Data analysis and statistics

The data were organized in Microsoft Excel spreadsheets and analyzed using the JASP statistical software (version 0.19.1.0; Amsterdam, Netherlands). Descriptive statistics were used to characterize the academic and professional profiles of the participating judges. Responses to the questionnaire items were presented as absolute (n) and relative (%) frequencies.

Content validation was conducted based on three parameters: the Content Validity Index (CVI), the Content Validity Coefficient (CVC), and the exact binomial test. The CVI was calculated for each item (Item-Level Content Validity Index - I-CVI), representing the proportion of judges who selected the highest categories on the Likert scale (e.g., “Agree” or “Totally agree”), divided by the total number of judges. For each phase of the instrument, according to the ADDIE model structure, the Scale-Level Content Validity Index (Average Calculation Method - S-CVI/AVE) was calculated by averaging the I-CVI values of the items pertaining to that phase^(13,14)^.

The CVC was also calculated for each phase of the instrument based on the items corresponding to each stage of evaluation. First, the item-level CVC (CVCi) was calculated by dividing the mean score assigned by the judges (∑x/j) by the maximum possible score on the Likert scale (Vmax). Next, the total CVC for the phase (CVCt) was obtained by averaging the CVCi values for the respective items and subtracting the standard error of judge polarization (PEj)^(15)^.

Additionally, the exact binomial test was applied to verify the statistical significance of agreement among the judges for each item. This test, appropriate for small samples, was conducted considering an expected agreement proportion of 0.80 and a significance level of 5% (p > 0.05), under the null hypothesis of judge agreement. Its use aimed to complement the CVI and CVC indices by estimating the statistical consistency of the evaluations, as adopted in previous studies^(16-18)^. CVI and CVC values were considered acceptable when greater than 0.80, indicating sufficient agreement among the judges^(13,15)^.

## RESULTS

Regarding the academic and professional profile of the judges who validated the course, 100% were nurses; 80% held a master’s degree; 80% held a doctoral degree; 90% worked as instructors, facilitators, or tutors in teaching and learning activities related to health, nursing, and/or entrepreneurship; 80% conducted and/or participated in scientific research in their field; 90% had published an article, book, or book chapter in their area of expertise; and 100% had over two years of professional experience in their field of practice, thereby meeting the selection criteria based on Fehring’s scoring system^(12)^.

The validation process carried out by the expert judges for the *Introduction to Entrepreneurship and Innovation in Nursing and Health Course* is presented in Tables 1 and 2, which display the scores assigned by the judges for each phase and the respective items used to assess the course’s structural and content validity. All items achieved CVI and CVC values above 0.80, indicating a high level of agreement among the judges and confirming the course’s validity across all evaluated aspects. The binomial test yielded p-values greater than 0.05, supporting the null hypothesis and reinforcing the statistical reliability of the CVI and CVC results. The mean agreement rates (I-CVI/Ave) were 99.0% for the Analysis phase, 100% for the Design phase, 100% for the Development phase, 99.0% for the Implementation phase, and 100% for the Evaluation phase. The total CVC (CVCt) for the phases ranged from 91.0% to 96.0%.

**Table 1:** Judges’ agreement for Phase 1, Analysis, and Design of the “Introduction to Entrepreneurship and Innovation in Nursing and Health” course, Chapecó, Santa Catarina, Brazil, 2024 (N = 10)

Assessed phases	Agreement n (%)			I- CVI	CVC_i_	P
	Disagree	Partially agree	Totally agree			
**Phase 1: Analysis**						
1. Relevance of the diagnosis used in the instructional planning	-	-	10 (100)	1.0	1.0	0.107
2. Identification of problems and critical points guiding the development of educational actions	-	-	10 (100)	1.0	1.0	0.107
3. Nature of the problem	1 (10)	1 (10)	8 (80)	0.90	0.90	0.376
4. Context in which the problem is situated	-	3 (30)	7 (70)	1.0	0.90	0.107
5. Target group performance	-	3 (30)	7 (70)	1.0	0.90	0.107
6. Types of educational strategies and technologies used	-	5 (50)	5 (50)	1.0	0.83	0.107
7. Adequacy of target audience definition	-	-	10 (100)	1.0	1.0	0.107
8. Course schedule - Adequacy of the course workload and delivery format	-	2 (20)	8 (80)	1.0	0.90	0.107
**(S-CVI/Ave)**	**0.99%**					
**CVC_t_**	**0.91%**					
**Phase 2: Design**	**n (%)**	**n (%)**	**n (%)**			
1. Definition of learning objectives - General objective	-	2 (20)	8 (80)	1.0	0.93	0.107
2. Definition of learning objectives - Specific objective 1	-	1 (10)	9 (90)	1.00	0.97	0.107
3. Definition of learning objectives - Specific objective 2	-	1 (10)	9 (90)	1.00	0.97	0.107
4. Learning plan development based on the ADDIE instructional model - General information	-	3 (30)	7 (70)	1.00	0.90	0.107
5. Learning plan development based on the ADDIE instructional model - Pedagogical content	-	3 (30)	7 (70)	1.00	0.90	0.107
6. Course structure design	-	4 (40)	6 (60)	1.00	0.87	0.107
7. Creation of a detailed lesson plan	-	3 (30)	7 (70)	1.00	0.90	0.107
8. Presentation of course content	-	3 (30)	7 (70)	1.00	0.90	0.107
9. Presentation of support materials	-	4 (40)	6 (60)	1.00	0.87	0.107
10. Presentation of the course structure	-	4 (40)	6 (60)	1.00	0.87	0.107
11. Presentation of participation prerequisites	-	2 (20)	8 (80)	1.00	0.93	0.107
12. Level 1 - Entrepreneurship applied to nursing. Topic 1 - Work in health and nursing	-	1 (10)	9 (90)	1.00	0.97	0.107
13. Level 1 - Entrepreneurship applied to nursing. Topic 2 - Concepts of entrepreneurship and innovation	-	-	10 (100)	1.00	1.00	0.107
14. Level 1 - Entrepreneurship applied to nursing. Topic 3 - Types of nursing entrepreneurship	-	-	10 (100)	1.00	1.00	0.107
15. Level 1 - Entrepreneurship applied to nursing. Topic 4 - Legislation	-	-	10 (100)	1.00	1.00	0.107
16. Level 2 - Entrepreneurship and innovation in nursing: practical aspects. Topic 1 - Exploring the idea	-	-	10 (100)	1.00	1.00	0.107
17. Level 2 - Entrepreneurship and innovation in nursing: practical aspects. Topic 2 - Pitching the idea and finding partners	-	-	10 (100)	1.00	1.00	0.107
18. Level 2 - Entrepreneurship and innovation in nursing: practical aspects. Topic 3 - Developing the idea	-	-	10 (100)	1.00	1.00	0.107
19. Level 2 - Entrepreneurship and innovation in nursing: practical aspects. Topic 4 - Protecting the idea	-	-	10 (100)	1.00	1.00	0.107
20. Level 2 - Entrepreneurship and innovation in nursing: practical aspects. Topic 5 - Self-development	-	-	10 (100)	1.00	1.00	0.107
**(S-CVI/Ave)**	**1.00%**					
**CVC_t_**	**0.96%**					

*A hyphen (-) indicates a numerical value of 0 not resulting from rounding; I-CVI - Item-Level Content Validity Index; CVC_i_ - Item-Level Content Validity Coefficient; CVC_t_ - Total Content Validity Coefficient; S-CVI/Ave - Scale-Level Content Validity Index using the Average method.*

**Table 2:** Judges’ agreement regarding the development, implementation, and evaluation of the “Entrepreneurship and Innovation in Nursing and Health” course, Chapecó, Santa Catarina, Brazil, 2024 (N = 10)

Assessed phases	Agreement n (%)			I- CVI	CVC_i_	P
	Disagree	Partially agree	Totally agree			
**Phase 3: Development**						
1. Development of course content	-	3 (30)	7 (70)	1.00	0.90	0.107
2. Definition of instructional strategies	-	2 (20)	8 (80)	1.00	0.93	0.107
3. Selection of instructional tools and technologies	-	2 (20)	8 (80)	1.00	0.93	0.107
4. Planning of learning activities	-	1 (10)	9 (90)	1.00	0.97	0.107
5. Definition of the instructional format	-	-	10 (100)	1.00	1.00	0.107
6. Definition of assessment methods	-	-	10 (100)	1.00	1.00	0.107
7. Coherence review	-	-	10 (100)	1.00	1.00	0.107
8. Assessment of the alignment between content and learning objectives	-	-	10 (100)	1.00	1.00	0.107
9. Course instructor presentation	-	-	10 (100)	1.00	1.00	0.107
**(S-CVI/Ave)**	**1.00%**					
**CVC_t_**	**0.96%**					
**Phase 4: Implementation**						
1. Course launch	-	4 (40)	6 (60)	1.00	0.87	0.107
2. Student registration process	-	3 (30)	7 (70)	1.00	0.90	0.107
3. Welcome and course introduction	-	2 (20)	8 (80)	1.00	0.93	0.107
4. Student orientation	-	2 (20)	8 (80)	1.00	0.93	0.107
5. Course management	-	2 (20)	8 (80)	1.00	0.93	0.107
6. Provision of physical infrastructure	1 (10)	2 (20)	7 (70)	0.90	0.87	0.376
7. Provision of administrative support	-	2 (20)	8 (80)	1.00	0.93	0.107
8. Provision of technological infrastructure	-	2 (20)	8 (80)	1.00	0.93	0.107
9. Identification and presentation of course facilitators	-	2 (20)	8 (80)	1.00	0.93	0.107
10. Identification and presentation of communication coordinators	-	3 (30)	7 (70)	1.00	0.90	0.107
11. Course coordination activities	-	4 (40)	6 (60)	1.00	0.87	0.107
**(S-CVI/Ave)**	**0.99%**					
**CVC_t_**	**0.92%**					
**Phase 5: Evaluation**						
1. Student performance evaluation	-	2 (20)	8 (80)	1.00	0.93	0.107
2. Formative assessments	-	2 (20)	8 (80)	1.00	0.93	0.107
3. Summative assessment	-	2 (20)	8 (80)	1.00	0.93	0.107
**(S-CVI/Ave)**	**1.00%**					
**CVC_t_**	**0.93%**					

*A hyphen (-) indicates a numerical value of 0 not resulting from rounding; I-CVI - Item-Level Content Validity Index; CVCi - Item-Level Content Validity Coefficient; CVCt - Total Content Validity Coefficient; S-CVI/Ave - Scale-Level Content Validity Index using the Average method.*

Agreement among the judges was reached in the first round of evaluation, eliminating the need for a second round, as demonstrated by the reported indices (Table 1). Although the course was validated in the first round, the comments provided were considered for improving future editions, as they did not compromise the structure or content of the course. Table 1 presents the level of agreement among the judges regarding the Analysis and Design phases of the course.

The following are the judges’ comments for each of the evaluated phases. In Phase 1, regarding the nature of the problem, Judge 1 highlighted the shortage of experienced and qualified instructors in the field of entrepreneurship.

*I would also point out the lack of instructors and professionals with well-developed skills acquired through experience and/or formal training in the field. This is essential for more nurses and/or nursing students to develop such skills by learning from peers who have already established a foothold in these settings.* (Judge 1)

Judge 9 emphasized the curricular focus on clinical nursing practice and noted that Brazil’s cultural orientation toward the Consolidation of Labor Laws (CLT) model, along with limited financial education in nursing training, represent key barriers.

*I believe the required nursing curriculum emphasizes the clinical role of nurses, relegating entrepreneurship to a secondary position. In addition, Brazilian culture does not cultivate self-driven success and is rooted in the CLT employment model. Entrepreneurship is about shifting one’s mindset. I think it’s essential to bring this brainstorming into the curriculum. Also, financial education-one of the foundations of entrepreneurship-should be included as a key topic.* (Judge 9)

Also in Phase 1, Judge 10 reported difficulties accessing the course platform and receiving support from the technical team.

*I’m a nursing professional who registered for the course; however, I was unable to access the course platform. I emailed the technical support team several times and never received a reply. I was really disappointed, as I was genuinely interested in taking the course.* (Judge 10)

At this stage, Judge 1 raised questions about the course’s target audience and the content of the “Developing Yourself” topic, requesting a more detailed description of these elements.

*As the target audience, does it include only nurses from the public healthcare system, or does it also include those from the private sector? Another question: does “Developing Yourself” refer to the entrepreneurial mindset as well? If yes to both, I fully agree with everything; if not, I believe those topics would also be valuable additions.* (Judge 1)

Also in Phase 2 (Design), Judge 9 suggested revising the course’s overall objective and clarifying the target audience, as well as incorporating content on artificial intelligence and financial literacy.

*Suggested main objective: “To educate students and health professionals to become entrepreneurs and innovators.” I also think it’s important to specify whether the course is for nursing, nurses, or health professionals in general. Introducing artificial intelligence is part of innovation and should be included in the theoretical framework. I also find the course lacks content on financial literacy and topics like “How to start your business” and “Your business in practice.”* (Judge 9)

Table 2 shows the level of agreement among the judges regarding the course’s development, implementation, and evaluation.

In both Phase 2 (Design) and Phase 3 (Development), Judge 9 highlighted the importance of including in-person sessions.

[...] *I suggest alternating between theoretical and practical classes, offered in both online and in-person formats.* [...] *I emphasize that interpersonal interaction is essential to promoting a mindset shift. I recommend holding the first session in person to facilitate networking and exposure to the entrepreneurial landscape.* (Judge 9)

In Phase 4 (Implementation), Judge 3 proposed offering the course in a hybrid format, including some in-person practical classes. In the same phase, Judge 9 recommended establishing a formal partnership with the Federal and Regional Nursing Councils (COFEN/COREN) to expand course outreach through their support networks.

Finally, in Phase 5 (Evaluation), Judges 3 and 9 encouraged the use of alternative assessment strategies.

*The assessment should involve the presentation of a business plan.* (Judge 3)

*As a summative evaluation, one suggestion would be to include a supplementary course in entrepreneurship* [e.g., courses from Sebrae]*, contact with* [or visits to] *entrepreneurs, or even the creation of a bootcamp for students to present a pitch.* (Judge 9)

## DISCUSSION

The *Entrepreneurship and Innovation in Nursing and Health Course* demonstrated strong structural and content validity. The high values obtained for the Content Validity Index and the Content Validity Coefficient, all above 0.80, indicate a high level of agreement among the judges and reinforce the perception that the course is appropriate in all evaluated aspects. These indices suggest that the content and its structural organization were broadly accepted and considered relevant by the evaluators, in alignment with methodological guidelines for validating health education courses^(18)^.

A detailed analysis of each course phase showed that the average agreement (I-CVI/Ave) remained above 99% across the board: 99.0% for the Analysis phase, 100% for Design, 100% for Development, 99.0% for Implementation, and 100% for Evaluation. This level of agreement suggests that the course was designed with a clear pedagogical structure and well-defined, accessible content, making it appropriate for teaching entrepreneurship and innovation in health and nursing. The consistently high indices across all phases reflect homogeneous agreement among the judges and confirm the course’s validity across multiple dimensions^(19)^.

Another important finding is that the total CVC ranged from 91.0% to 96.0% across phases, reinforcing the perception of high content validity. Recommended in the literature for content validation^(13,14)^, these indices show that the course material was highly rated in terms of relevance, clarity, and applicability. This finding is essential for the course’s acceptance in future cohorts, as it ensures that the program meets the necessary quality standards for teaching in nursing and health contexts.

Entrepreneurship is a dynamic process that requires initiative from health professionals. However, there is ongoing debate regarding the extent to which entrepreneurial characteristics can be taught, as both personal and contextual factors have a direct influence on entrepreneurial profiles. Elements such as having entrepreneurial parents or previous work experience can significantly affect the development of entrepreneurial competencies and hinder the measurement of educational outcomes in this area^(20)^. This concern aligns with the comment from Judge 1, who highlighted the shortage of faculty and professionals with formal training in entrepreneurship, the negative implications of this gap for training entrepreneurial nurses, and the need to foster such competencies among practicing professionals.

The agreement among the judges in the first round of evaluation further highlights the effectiveness of the methodology adopted in the course design. Although a second round was not required, the judges’ suggestions were considered to enhance future editions. The absence of significant modifications to the course structure or content, combined with the initial agreement, allowed the course to be validated after the first round. This underscores the positive impact of the planning and development process, which took into account local gaps and needs, as emphasized by Judge 9.

The suggestions and comments made throughout the different phases reinforce that the course is designed to encourage nurses to develop a critical perspective on their professional practice. The course content highlights the need for training and qualification in areas that go beyond technical and clinical care, addressing broader aspects of care delivery. This includes tools to foster client engagement and connection, as well as strategies to overcome challenges related to bureaucracy, business management, financial planning, service pricing, and client acquisition^(6)^-aspects that the participants valued.

In contrast, critics of entrepreneurial education in Brazil argue that it can promote entrepreneurship as a normative model that induces students to desire self-employment and risk taking, while placing the burden of training, success, or failure solely on the individual. They claim that this model may represent a form of pedagogy that limits alternative understandings of education as a citizen’s right and a public good^(21)^. Others argue that entrepreneurship may become an ideological tool used to reinforce the sociometabolic reproduction of capital^(22)^. Nonetheless, it is important to emphasize that the current expanded perspective on entrepreneurship as an educational tool for competency development extends beyond professional performance. It reaches into the realm of purpose, relationship-building, and value creation for the client.

### Study limitations

This study has some limitations inherent to the method adopted. The first refers to the small number of participating judges, which may compromise the generalizability of the results and affect the statistical robustness of the content validation. Although all items met the established agreement criteria, the small sample size limits the reliability of the findings. Additionally, there is a possibility of subjective bias in the judges’ assessments-that is, they may have been influenced by their personal experiences and professional backgrounds, leading to individual interpretations of the instrument’s items. While the findings indicate the course’s structural and content validity, its quality can be continually improved with each new edition through complementary approaches, such as including a larger number of judges, gathering more detailed qualitative feedback, and assessing its impact on professional practice.

### Contributions to the field of Nursing, Health, or Public Policy

The *Introduction to Entrepreneurship and Innovation in Nursing and Health Course* emerges as a validated educational resource for nursing and health practice. It meets the pedagogical needs of its intended context and supports training that is better aligned with the growing demands for innovation and management in healthcare. The course contributes to the nursing curriculum-and that of other professionals who benefit from it-by expanding the settings for professional engagement and practice. It serves as a tool to strengthen professional competencies, foster more autonomous and innovative practice, and prepare professionals to meet contemporary challenges.

## FINAL CONSIDERATIONS

The *Entrepreneurship and Innovation in Nursing and Health Course* demonstrated high structural and content validity, showing both theoretical and methodological soundness, as well as potential for replication and for promoting entrepreneurial training in nursing and health. It helps establish a new professional outlook focused on innovation, continuous planning, and creating professional value. Moreover, it enhances access to training resources for entrepreneurship in nursing and health and broadens both visibility and career opportunities.

## Data Availability

The research data are available in a repository: https://doi.org/10.17605/OSF.IO/PCSYB.
