# Supplementary material for: Validation of an online course on introduction to entrepreneurship and innovation in nursing and health
Source: Rev Bras Enferm. 2025 Dec 8;78(6):e20240614. doi: 10.1590/0034-7167-2024-0614 (PMC12707530; doi:10.1590/0034-7167-2024-0614)
Supplement: Translation

# Translation: Validação do curso virtual de introdução ao empreendedorismo e inovação em enfermagem e saúde

**DOI:** 10.1590/0034-7167-2024-0614pt

**Authors:** Jouhanna do Carmo Menegaz, Aurilívia Carolinne Lima Barros, Letícia de Lima Trindade, Thais de Fátima Aleixo Corrêa, Matheus Moraes Silva, Emily Silva Pontes

**Affiliations:** I Universidade do Estado de Santa Catarina. Chapecó, Santa Catarina, Brasil; II Barros Tecnologia LTDA. Caxias, Maranhão, Brasil; III Faculdade Estácio do Pará. Belém, Pará, Brasil; IV Universidade Federal do Pará. Belém, Pará, Brasil; V Fundação Universitária Evangélica. Anápolis, Goiás, Brasil

**Keywords:** Empreendedorismo, Enfermagem, Estudo de Validação, Saúde, Pessoal de Saúde.

## Abstract

**Objetivos:**

validar um curso virtual de introdução ao empreendedorismo e inovação em enfermagem e saúde.

**Métodos:**

estudo metodológico para a validação estrutural e de conteúdo do “Curso de Introdução ao Empreendedorismo e Inovação em Enfermagem e Saúde”. Dez juízes participaram do processo de validação proposto e responderam ao questionário para avaliação da validade do curso. A validação utilizou os seguintes parâmetros: Índice de Validade de Conteúdo, Coeficiente de Validade de Conteúdo e o teste exato de distribuição binomial.

**Resultados:**

o curso foi validado na primeira rodada de avaliação. A falta de docentes e profissionais com habilidades empreendedoras desenvolvidas, a ênfase curricular na formação assistencial e questões logísticas do curso foram apontadas pelos juízes.

**Considerações Finais:**

os achados demonstram elevada validade de estrutura e conteúdo do curso, evidenciando qualidade teórica e metodológica, atendendo ao atual cenário, o qual exige acesso a programas que fomentem a formação empreendedora em Enfermagem e Saúde.

## INTRODUÇÃO

A compreensão do empreendedorismo perpassa por diversos conceitos, desde a exclusiva geração de lucros até definições relacionadas ao comportamento do empreendedor^(1)^. Independentemente dos conceitos, o empreendedor está inserido em um processo dinâmico e criativo de identificação e captação de oportunidades. Nesse contexto, ele diferencia-se ao adotar uma nova visão orientada à inovação, ao planejamento contínuo e à assunção de riscos calculados, visando agregar valor financeiro, científico e/ou tecnológico^(2)^.

Na enfermagem, o ato de empreender expande a atuação profissional, revela perspectivas do trabalho para além dos hospitais e outras unidades públicas e privadas de assistência à saúde, aumenta a visibilidade e as oportunidades por meio da ciência e da inovação, bem como preenche lacunas no atual sistema de saúde relacionadas ao desenvolvimento de produtos, treinamentos e serviços educacionais e de pesquisa^(3,4)^.

Em sua atuação, o enfermeiro usa seu conhecimento técnico-científico com foco em práticas baseadas em evidências e atualizações constantes; paralelamente, ele tem a capacidade de desenvolver e gerir ideias, negócios e produtos tecnológicos sustentáveis e eficientes^(5)^. Por um lado, a enfermagem possui a utilização técnico-científica das práticas cuidativas em seus alicerces. Por outro lado, tem desenvolvido um perfil empreendedor cada vez mais consolidado, que, embora distante de seu pleno potencial, já delineou sua trajetória no contexto nacional em diversas vertentes profissionais.

Diante do leque de possiblidades que o empreendedorismo proporciona aos enfermeiros, é necessário que seja estimulado; e os desafios, enfrentados. Muitos enfermeiros têm encontrado dificuldades em aderir a ele em sua prática profissional. Alguns custam a entender como as competências empreendedoras podem auxiliá-los a alavancar suas práticas profissionais bem como favorecer a qualidade do trabalho e a experiência do cliente. Outros, sobretudo os enfermeiros empreendedores, vivenciam barreiras relacionadas ao conhecimento insuficiente sobre os processos burocráticos, gestão de negócios, gestão financeira, precificação de seus serviços e captação de clientes, além da aceitação limitada da sociedade em reconhecer o profissional como empreendedor^(6)^.

Para enfrentar esses obstáculos, a educação empreendedora é essencial, pois prepara fornecendo conhecimentos específicos relacionados a várias áreas fragilizadas no processo de trabalho da enfermagem. Dado que o empreendedorismo envolve competências não contempladas pela estrutura curricular da maior parte das instituições de ensino de enfermagem brasileiras, é preciso preencher essas lacunas para que o profissional possa adentrar e se consolidar no mundo do empreendedorismo^(7)^.

O fomento à educação empreendedora e o incentivo à inovação são cruciais para a transformação de mentalidade por meio da ciência na enfermagem. Para isso, nas bases curriculares da profissão, é necessário incluir estruturas que apoiem a inovação em áreas como a educação, a prática e a pesquisa, assim como promover parcerias com outros profissionais e disciplinas que estimulem a resolução de problemas^(8)^.

Além do incentivo nos currículos acadêmicos, outras iniciativas podem ser utilizadas para introduzir conceitos específicos do empreendedorismo na vivência profissional. Os cursos extracurriculares promovem competências profissionais como planejamento de negócios e organização e gestão financeira, que são essenciais para os enfermeiros iniciarem seus próprios empreendimentos^(9)^.

Evidencia-se que a educação empreendedora na formação inicial pode proporcionar benefícios efetivos à área da saúde em seu conjunto. A sua inserção na educação permanente de profissionais propicia o desenvolvimento de competências empreendedoras nítidas e aplicáveis. Com isso, constrói-se no profissional características diferenciadas e importantes ao desenvolvimento de suas práticas, como proatividade, criticidade, vontade de inovar e planejar, autoconfiança, busca do agir baseado em evidências, capacidade de negociação e gestão de riscos, liderança e inter-relações, entre outras características^(10)^.

A escassez de oferta de cursos de educação empreendedora voltados para a enfermagem - e orientados a transpor as barreiras do conhecimento dentro dessa temática tão relevante e atual - estimulou a criação do “Curso de Introdução ao Empreendedorismo e Inovação em Enfermagem e Saúde”, a fim de apoiar o desenvolvimento de competências empreendedoras entre esses profissionais. Assim, a disponibilização do curso visa preencher a lacuna do conhecimento sobre empreendedorismo dentro da área de enfermagem e saúde, fornecendo arcabouço conceitual quanto atividades reflexivas e práticas.

Para qualificar o curso criado no tocante ao conteúdo e estrutura, propôs-se sua validação com especialistas na área de enfermagem a fim de oferecer futuramente uma versão atualizada e validada para enfermeiros e profissionais de saúde do Brasil e do mundo.

## OBJETIVOS

Validar um curso de introdução ao empreendedorismo e inovação em enfermagem e saúde.

## MÉTODOS

### Aspectos éticos

Este estudo seguiu as diretrizes de ética nacionais (Resolução n. 466/2012) e internacionais. Foi aprovado pelo Comitê de Ética em Pesquisa Envolvendo Seres Humanos, da Universidade do Estado de Santa Catarina, sob Parecer consubstanciado nº 5.440.353 CEP/UDESC. Todos os participantes assinaram o Termo de Consentimento Livre e Esclarecido (TCLE).

### Desenho, período e local do estudo

Trata-se de estudo metodológico para validar a estrutura e conteúdo do “Curso de Introdução ao Empreendedorismo e Inovação em Enfermagem e Saúde”. Foi realizado no formato on-line mediante preenchimento de formulário de pesquisa. O curso objetiva a capacitação de estudantes e profissionais da área da enfermagem. Ocorreu entre 2022 e 2023, conforme a estrutura do modelo *Analysis, Design, Development, Implementation, and Evaluation* (ADDIE), utilizado para criar programas educacionais e oferecido remotamente por meio do sistema *Massive Open Online Course* (MOOC) pela Universidade do Estado de Santa Catarina (UDESC). Sua validação seguiu as cinco fases desse modelo, ou seja, Análise, Desenho, Desenvolvimento, Implementação e Avaliação.

O protocolo da pesquisa está registrado na plataforma Open Science Framework (OSF.io) sob o DOI https://doi.org/10.17605/OSF.IO/PCSYB. Utilizou-se a lista de verificação SQUIRE^(11)^ para construir o relatório norteador da pesquisa.

### Amostra, critérios de inclusão e exclusão

Foram convidados a participar da validação do curso 15 juízes especialistas, que eram profissionais da área da enfermagem com experiência em gestão, assistência, ensino e produção científica relevante, conforme critérios de seleção estabelecidos por um instrumento construído mediante a adaptação do sistema de pontuação de Fehring^(12)^. A seleção respeitou os seguintes critérios: ser profissional na área da enfermagem e atender ao ponto de corte 5 no instrumento de seleção.

Entre os juízes convidados, cinco não responderam ao convite, sendo excluídos da pesquisa. Após a aplicabilidade dos critérios de Fehring^(12)^, todos os participantes que aceitaram participar da pesquisa superaram o ponto de corte, habilitando esses dez juízes a participarem do processo de validação proposto.

O instrumento de seleção contou com sete itens avaliativos. As questões relacionavam-se às titulações do profissional, desenvolvimento de pesquisa na área, produção intelectual, formação didático-pedagógica na área e prática profissional superior a dois anos, apresentando pontuação máxima de 12.

Quanto ao nível de atendimento aos critérios de seleção, 1 juiz atendeu em 11 pontos, 6 juízes atenderam em 9 pontos, 1 juiz em 8 pontos e 2 juízes em 5 pontos. O critério “Prática profissional de, pelo menos, dois anos em sua área de atuação” foi o mais evidenciado, sendo atendido por todos os dez juízes. Essa característica demonstra que eles têm experiência profissional em suas áreas de atuação. Além disso, os critérios “Publicação de artigo, livro ou capítulo de livro” (atendido por oito juízes) e “Professor de formação didático-pedagógica em saúde ou afim, TI ou Enfermagem ou afim” (atendido por sete juízes) evidenciam que possuem produção intelectual e acadêmica bem como experiência docente relevante. Dessa forma, pode-se inferir que esses juízes detêm uma base prática robusta para subsidiar suas avaliações.

### Protocolo do estudo

Os juízes responderam um questionário para mensurar a validade do curso, estruturado conforme modelo instrucional adotado, o modelo ADDIE. O questionário foi disponibilizado em formulário eletrônico disponibilizado por meio do aplicativo de mensagens WhatsApp. Com o resultado da resposta dos juízes, foi possível medir a validade do curso quanto à estrutura e conteúdo, conforme proposto no objetivo do estudo.

A construção do curso foi orientada pelas fases do modelo ADDIE. Dessa maneira, a avaliação seguiu o mesmo critério. O modelo apresenta cinco fases: Análise, Desenho, Desenvolvimento, Implementação e Avaliação.

- **Análise** - envolve o mapeamento da natureza do problema a ser enfrentado, o contexto de inserção do problema e o desempenho alvo das ações;
- **Desenho** - inclui a criação de um plano de aula detalhado, apresentando os conteúdos, materiais de suporte, a estrutura do curso, os pré-requisitos para a participação e a sequência adequada dos conteúdos para favorecer a aprendizagem;
- **Desenvolvimento** - nessa fase, o conteúdo do curso é criado, são definidas as estratégias de ensino, os recursos e ferramentas didáticos e tecnológicos, os exercícios e atividades de aprendizagem, bem como a forma de interação e as modalidades de avaliação;
- **Implementação** - o curso é implementado nessa fase;
- **Avaliação** - o desempenho dos alunos é avaliado para medir a eficácia do curso realizado, podendo ocorrer de forma diversificada com a inclusão de avaliações formativas e somativas.

Essas fases foram analisadas de acordo com o instrumento para validação, composto por sete seções. As duas primeiras correspondem ao Termo de Consentimento Livre e Esclarecido e às Instruções. Já as cinco seções seguintes direcionam-se à avaliação do curso conforme as fases do modelo ADDIE, que conduziu a construção do curso e norteou a estrutura de validação. Tais critérios serviram como guia desde o início do desenvolvimento da proposta até sua avaliação. Essa abordagem garantiu a coerência entre a estrutura instrucional construída e os parâmetros utilizados para sua validação.

Cada seção temática do instrumento de avaliação conteve questões de múltipla escolha, na forma de Escala Likert de quatro pontos, e uma questão discursiva opcional ao final da seção para registrar sugestões, orientações ou justificativas relacionadas às respostas anteriores. A Escala foi constituída dos seguintes parâmetros: Discordo, Concordo parcialmente e Concordo totalmente. Os blocos de questões relacionaram-se às fases do modelo ADDIE.

Na fase de Análise, foram mapeados os problemas, o contexto de inserção do problema, o desempenho-alvo das ações, os tipos de processos educacionais a serem utilizados, a definição do público-alvo e os pontos críticos que demandam ações educativas.

No Desenho, foi observada a elaboração de um plano de aula detalhado, a apresentação dos conteúdos, os materiais de suporte e a estrutura do curso.

Na fase de Desenvolvimento, em que o conteúdo do curso é criado, foram avaliadas as estratégias de ensino, os recursos, as ferramentas didáticas e tecnológicas, os exercícios e atividades de aprendizagem, bem como a forma de interação e as modalidades de avaliação. Nessa fase, também houve a seleção dos docentes para cada atividade desenvolvida.

A Implementação contemplou as ações de acolhimento e instrução, a orientação dos alunos e a administração do curso.

Por fim, na fase de Avaliação, o desempenho dos alunos foi avaliado para medir a eficácia do curso realizado. A avaliação foi realizada de forma diversificada, envolvendo avaliações formativas e somativas.

### Análise dos dados e estatística

Os dados foram organizados em planilhas do Microsoft Excel e analisados com auxílio do software estatístico Jasp (versão 0.19.1,0; Holanda, Amsterdã). Foram realizadas estatísticas descritivas para caracterizar o perfil acadêmico-profissional dos juízes participantes. As respostas aos itens do instrumento de avaliação foram apresentadas em termos de frequência absoluta (n) e relativa (%).

A validação de conteúdo foi efetuada com base em três parâmetros: o Índice de Validade de Conteúdo (CVI), o Coeficiente de Validade de Conteúdo (CVC) e o teste exato de distribuição binomial. O CVI foi calculado para cada item (*Item-Level Content Validity Index -* I-CVI), representando a proporção de juízes que atribuíram aos itens as maiores categorias da escala Likert (p.ex., “Concordo” ou “Concordo totalmente”), dividida pelo número total de juízes. Para cada uma das fases do instrumento, conforme estrutura do modelo ADDIE, foi calculado o Índice de Validade de Conteúdo da Escala (Scale-Level Content Validity Index, Average Calculation Method - S CVI/AVE), obtido pela média dos I-CVI dos itens pertencentes à respectiva fase^(13,14)^.

O CVC também foi calculado para cada fase do instrumento, considerando os itens correspondentes a cada etapa de avaliação. Inicialmente, calculou-se o CVC de cada item (CVCi) por meio da média das pontuações atribuídas pelos juízes (∑x/j), dividida pelo valor máximo da escala Likert (Vmáx). Em seguida, o CVC total da fase (CVCt) foi obtido pela média dos CVCi dos itens daquela fase, subtraída do erro-padrão da polarização dos juízes (PEj)^(15)^.

Adicionalmente, foi aplicado o teste exato de distribuição binomial para verificar a significância estatística da concordância entre os juízes para cada item do instrumento. Esse teste, indicado para pequenas amostras, foi conduzido considerando uma proporção esperada de concordância de 0,80 e nível de significância de 5% (p > 0,05), sob a hipótese nula de que os juízes estariam em concordância. Sua aplicação buscou complementar os índices CVI e CVC, estimando a consistência estatística das avaliações, conforme utilizado em estudos anteriores^(16-18)^. Valores de CVI e CVC foram considerados aceitáveis quando superiores a 0,80, refletindo concordância entre os juízes^(13,15)^.

## RESULTADOS

Quanto à caracterização do perfil acadêmico-profissional dos juízes que validaram o curso, 100% eram enfermeiros; 80%, mestres; 80%, doutores; 90% atuavam como professor, facilitador ou tutor na formação didático-pedagógica em saúde, enfermagem e/ou empreendedorismo; 80% realizavam e/ou participavam de pesquisas científicas em sua área de atuação; 90% publicaram artigo, livro ou capítulo de livro em sua área de atuação; 100% atuavam há mais de dois anos em sua área de formação, atendendo assim ao sistema de pontuação de Fehring^(12)^ adotado para a seleção.

O processo de julgamento dos juízes-especialistas quanto à validade do “Curso de Empreendedorismo e Inovação em Enfermagem e Saúde” é apresentado nas Tabelas 1 e 2, em que constam os valores atribuídos pelos juízes para cada fase e seus respectivos itens para a avaliação da validade da estrutura e conteúdo do curso. Todos os itens obtiveram CVI e CVC superiores a 0,80, demonstrando alta concordância entre os juízes e validade do curso quanto a todos os aspectos avaliados. O teste binomial apresentou valores superiores a *p* > 0,05, confirmando a hipótese nula e fornecendo confiabilidade estatística ao CVI e CVC. A média de concordância (I-CVI/Ave) foi de 99,0% para a fase de Análise, 100% para Desenho, 100% para Desenvolvimento, 99,0% para Implementação e 100% para Avaliação. O CVCt das fases variou entre 91,0% e 96,0%.

**Tabela 1:** Concordância dos juízes para a Fase 1, análise e desenho do “Curso de Empreendedorismo e Inovação em Enfermagem e Saúde”, Chapecó, Santa Catarina, Brasil, 2024 (N = 10)

Fases avaliadas	Concordância %			I- CVI	CVC_i_	P
	Discordo	Concordo parcialmente	Concordo totalmente			
**Fase 1: Análise**						
1. Pertinência no delineamento do diagnóstico situacional	-	-	10 (100)	1,0	1,0	0,107
2. Identificação de problemas e pontos críticos que demandem ações educativas	-	-	10 (100)	1,0	1,0	0,107
3. Natureza do problema	1 (10)	1 (10)	8 (80)	0,90	0,90	0,376
4. Contexto de inserção do problema	-	3 (30)	7 (70)	1,0	0,90	0,107
5. Desempenho-alvo das ações	-	3 (30)	7 (70)	1,0	0,90	0,107
6. Tipo de processos educacionais utilizados	-	5 (50)	5 (50)	1,0	0,83	0,107
7. Adequação da definição do público-alvo	-	-	10 (100)	1,0	1,0	0,107
8. Cronograma - Adequação da carga horária e período de disponibilização do curso	-	2 (20)	8 (80)	1,0	0,90	0,107
**(S-CVI/Ave)**	**0,99%**					
**CVC_t_**	**0,91%**					
**Fase 2: Desenho**	**n (%)**	**n (%)**	**n (%)**			
1. Definição dos objetivos de aprendizagem - Objetivo geral	-	2 (20)	8 (80)	1,0	0,93	0,107
2. Definição dos objetivos de aprendizagem - Objetivo específico 1	-	1 (10)	9 (90)	1,00	0,97	0,107
3. Definição dos objetivos de aprendizagem - Objetivo específico 2	-	1 (10)	9 (90)	1,00	0,97	0,107
4. Projeto do plano de aprendizagem construído conforme modelo instrucional ADDIE - Dados gerais	-	3 (30)	7 (70)	1,00	0,90	0,107
5. Projeto do plano de aprendizagem construído conforme modelo instrucional ADDIE - Conteúdos pedagógicos	-	3 (30)	7 (70)	1,00	0,90	0,107
6. Desenho da estrutura do curso	-	4 (40)	6 (60)	1,00	0,87	0,107
7. Criação de um plano de aula detalhado	-	3 (30)	7 (70)	1,00	0,90	0,107
8. Apresentação dos conteúdos	-	3 (30)	7 (70)	1,00	0,90	0,107
9. Apresentação dos materiais de suporte	-	4 (40)	6 (60)	1,00	0,87	0,107
10. Apresentação da estrutura do curso	-	4 (40)	6 (60)	1,00	0,87	0,107
11. Apresentação dos pré-requisitos para a participação	-	2 (20)	8 (80)	1,00	0,93	0,107
12. Nível 1 - Empreendedorismo aplicado à Enfermagem. Tópico 1 - Trabalho em saúde e Enfermagem	-	1 (10)	9 (90)	1,00	0,97	0,107
13. Nível 1 - Empreendedorismo aplicado à Enfermagem. Tópico 2 - Conceitos de empreendedorismo e inovação	-	-	10 (100)	1,00	1,00	0,107
14. Nível 1 - Empreendedorismo aplicado à Enfermagem. Tópico 3 - Tipologias de empreendedorismo de Enfermagem	-	-	10 (100)	1,00	1,00	0,107
15. Nível 1 - Empreendedorismo aplicado à Enfermagem. Tópico 4 - Legislação	-	-	10 (100)	1,00	1,00	0,107
16. Nível 2 - Empreendendo e inovando em Enfermagem: aspectos práticos. Tópico 1 - Explorando a ideia	-	-	10 (100)	1,00	1,00	0,107
17. Nível 2 - Empreendendo e inovando em Enfermagem: aspectos práticos. Tópico 2 - Vendendo a ideia e encontrando parceiros	-	-	10 (100)	1,00	1,00	0,107
18. Nível 2 - Empreendendo e inovando em Enfermagem: aspectos práticos. Tópico 3 - Desenvolvendo a ideia	-	-	10 (100)	1,00	1,00	0,107
19. Nível 2 - Empreendendo e inovando em Enfermagem: aspectos práticos. Tópico 4 - Registrando a ideia	-	-	10 (100)	1,00	1,00	0,107
20. Nível 2 - Empreendendo e inovando em Enfermagem: aspectos práticos. Tópico 5 - Desenvolvendo a si mesmo	-	-	10 (100)	1,00	1,00	0,107
**(S-CVI/Ave)**	**1,00%**					
**CVC_t_**	**0,96%**					

*O hífen (-) significa dado numérico igual a 0 não resultante de arredondamento; I-CVI - Item-Level Content Validity Index; CVC_i_ - Coeficiente de Validade de Conteúdo por item; CVCt - Coeficiente de Validade de Conteúdo total; S-CVI/Ave - Scale-Level Content Validity Index/Average Calculation Method.*

**Tabela 2:** Concordância dos juízes quanto ao desenvolvimento, implementação e avaliação do “Curso de Empreendedorismo e Inovação em Enfermagem e Saúde”, Chapecó, Santa Catarina, Brasil, 2024. (N = 10)

Fases avaliadas	Concordância n (%)			I- CVI	CVC_i_	P
	Discordo	Concordo parcialmente	Concordo totalmente			
**Fase 3: Desenvolvimento**						
1. Criação do conteúdo do curso	-	3 (30)	7 (70)	1,00	0,90	0,107
2. Definição das estratégias de ensino	-	2 (20)	8 (80)	1,00	0,93	0,107
3. Definição dos recursos e ferramentas didáticos e tecnológicos	-	2 (20)	8 (80)	1,00	0,93	0,107
4. Definição dos exercícios e atividades de aprendizagem	-	1 (10)	9 (90)	1,00	0,97	0,107
5. Definição da forma de interação	-	-	10 (100)	1,00	1,00	0,107
6. Definição das modalidades de avaliação	-	-	10 (100)	1,00	1,00	0,107
7. Avaliação da coerência	-	-	10 (100)	1,00	1,00	0,107
8. Avaliação do alinhamento do conteúdo com os objetivos	-	-	10 (100)	1,00	1,00	0,107
9. Apresentação do coordenador do curso	-	-	10 (100)	1,00	1,00	0,107
**(S-CVI/Ave)**	**1,00%**					
**CVC_t_**	**0,96%**					
**Fase 4: Implementação**						
1. Lançamento do curso	-	4 (40)	6 (60)	1,00	0,87	0,107
2. Início da participação dos alunos no curso	-	3 (30)	7 (70)	1,00	0,90	0,107
3. Ações de acolhimento e instrução	-	2 (20)	8 (80)	1,00	0,93	0,107
4. Orientação dos alunos	-	2 (20)	8 (80)	1,00	0,93	0,107
5. Administração do curso	-	2 (20)	8 (80)	1,00	0,93	0,107
6. Provimento de estrutura física	1 (10)	2 (20)	7 (70)	0,90	0,87	0,376
7. Provimento de estrutura administrativa	-	2 (20)	8 (80)	1,00	0,93	0,107
8. Provimento de estrutura tecnológica	-	2 (20)	8 (80)	1,00	0,93	0,107
9. Definição e apresentação dos moderadores	-	2 (20)	8 (80)	1,00	0,93	0,107
10. Definição e apresentação dos responsáveis pela comunicação	-	3 (30)	7 (70)	1,00	0,90	0,107
11. Atividades de coordenação	-	4 (40)	6 (60)	1,00	0,87	0,107
**(S-CVI/Ave)**	**0,99%**					
**CVC_t_**	**0,92%**					
**Fase 5: Avaliação**						
1. Avaliação do desempenho dos alunos	-	2 (20)	8 (80)	1,00	0,93	0,107
2. Avaliações formativas	-	2 (20)	8 (80)	1,00	0,93	0,107
3. Avaliações somativas	-	2 (20)	8 (80)	1,00	0,93	0,107
**(S-CVI/Ave)**	**1,00%**					
**CVC_t_**	**0,93%**					

*O hífen (-) significa dado numérico igual a 0 não resultante de arredondamento; I-CVI - Item-Level Content Validity Index; CVC_i_ - Coeficiente de Validade de Conteúdo por item; CVCt - Coeficiente de Validade de Conteúdo total; S-CVI/Ave - Scale-Level Content Validity Index/Average Calculation Method.*

A concordância entre os juízes foi alcançada na primeira rodada de avaliação, dispensando uma segunda rodada de avaliação conforme evidenciado pelos índices apresentados (Tabela 1). Embora o curso tenha alcançado a validação na primeira rodada, as sugestões registradas nos comentários foram consideradas para aprimorar futuras edições do curso, uma vez que não comprometeram a estrutura nem o conteúdo da proposta. Na Tabela 1, apresenta-se o grau de concordância dos juízes quanto à análise e desenho do curso.

A seguir, são apresentados os comentários dos juízes para cada uma das fases avaliadas. Na Fase 1, quanto à natureza do problema, o Juiz 1 destacou a falta de docentes experientes e habilitados na área do empreendedorismo.

*Destaco também a falta de docentes e profissionais com habilidades desenvolvidas em decorrência da experiência e/ou formação na área para que mais enfermeiros e/ou futuros enfermeiros desenvolvam essas habilidades a partir da percepção de colegas que conquistaram seus espaços nesses contextos*. (Juiz 1)

O Juiz 9 apontou a ênfase da estrutura curricular na formação assistencial do enfermeiro, ressaltando o direcionamento da cultura brasileira para o regime da Consolidação das Leis do Trabalho (CLT) e a deficiência da educação financeira na formação.

*Acredito que a grade curricular obrigatória na área de enfermagem enfatiza a ênfase do enfermeiro assistencial, deixando o empreendedorismo para um segundo plano, além da cultura brasileira não cultivar o autossucesso e ser baseada no regime CLT! O empreendedorismo está voltado para a mudança de mindset. Acho superimportante trazer esse brainstorming para a grade curricular. Lembrar que uma das bases do empreendedorismo é a educação financeira, que deve ser inserida em um dos assuntos a ser abordado também.* (Juiz 9)

Ainda na Fase 1, foi apontado pelo Juiz 10 a dificuldade de acesso à plataforma do curso e de contato com o suporte técnico.

*Eu fui uma profissional de enfermagem que tinha feito inscrição para o curso; entretanto, não consegui acessar a plataforma do curso! Por várias vezes, enviei e-mail para o suporte técnico e não recebi retorno. Fiquei um pouco triste com a situação, pois tinha total interesse em realizar o curso.* (Juiz 10)

Nessa fase, o Juiz 1 questionou sobre o público-alvo do curso e conteúdo do tópico “Desenvolvendo a si mesmo”, solicitando uma descrição mais detalhada desses itens.

*Como público-alvo, inclui enfermeiros da rede pública apenas ou a privada se inclui também? Outra dúvida: o “desenvolvendo a si mesmo” diz respeito à mentalidade empreendedora também? Se sim para ambos, concordo totalmente com tudo, se não, acredito que esses tópicos também seriam de grande contribuição.* (Juiz 1)

Ainda na Fase 2 (Desenho), o Juiz 9 sugeriu modificar o objetivo geral do curso e definir melhor o público-alvo, além de introduzir conteúdos como inteligência artificial e educação financeira.

*Sugestão para o objetivo principal: “Educar alunos e profissionais da saúde para empreender e inovar”. Também acho que precisa de uma definição se é para enfermagem, enfermeiros ou profissionais da saúde. A introdução à inteligência artificial faz parte da inovação e deve fazer parte da grade teórica. Sinto falta da educação financeira e tópicos: “Como montar seu negócio”, “seu negócio na prática*”. (Juiz 9)

Na Tabela 2, apresenta-se o grau de concordância dos juízes quanto ao desenvolvimento, implementação e avaliação do curso.

Tanto na Fase 2 (Desenho) quanto na Fase 3 (Desenvolvimento), o Juiz 9 ressaltou a importância de encontros presenciais.

[...] *sugiro intercalar aulas teóricas e práticas, entre on-line e presencial.* [...] *Saliento que o contato interpessoal é importante para a mudança de mindset. Sugiro que a primeira aula seja presencial, para gerar networking e conhecimento do mercado empreendedor.* (Juiz 9)

Também na Fase 4 (Implementação), o Juiz 3 propôs a disponibilização do curso na modalidade híbrida, com algumas aulas práticas presenciais. Ainda nessa fase, o Juiz 9 sugeriu a formalização de parceria com o sistema Conselho Federal de Enfermagem/Conselhos Regionais (COFEN/COREN), para abranger um público maior mediante a divulgação em suas redes de apoio.

Por fim, na Fase 5, os Juízes 3 e 9 estimularam a utilização de formas alternativas de avaliação.

*A avaliação deveria ser a apresentação de um plano de negócio.* (Juiz 3)

*Como avaliação somativa, uma sugestão seria incluir um curso extra em empreendedorismo* [p.ex., cursos do Sebrae], *contato com* [ou visita a] *algum empreendedor ou até mesmo o desenvolvimento de um bootcamp para apresentação de um pitch do aluno.* (Juiz 9)

## DISCUSSÃO

O “Curso de Empreendedorismo e Inovação em Enfermagem e Saúde” demonstrou elevada validade de estrutura e conteúdo. Os valores elevados para o Índice de Validade de Conteúdo e para o Coeficiente de Validade de Conteúdo - todos acima de 0,80 - indicam um alto nível de concordância entre os juízes e reforçam a percepção de que o curso é adequado quanto aos aspectos avaliados. Esses índices sugerem que o conteúdo abordado e sua organização estrutural foram amplamente aceitos e considerados pertinente pelos avaliadores, alinhando-se com as diretrizes metodológicas para a validação de cursos voltados à educação em saúde^(18)^.

A análise detalhada das fases do curso mostra que a média de concordância (I-CVI/Ave) manteve-se acima de 99% em todas as etapas: Análise (99,0%), Desenho (100%), Desenvolvimento (100%), Implementação (99,0%) e Avaliação (100%). Esse nível de consenso sugere que o curso foi elaborado com uma estrutura pedagógica clara e conteúdos bem delimitados e acessíveis, sendo adequado para a proposta de ensino do empreendedorismo e inovação na área da saúde e enfermagem. As fases apresentaram índices consistentes, refletindo uma aceitação homogênea entre os juízes e validando o curso em múltiplas dimensões^(19)^.

Outro aspecto relevante é que o CVC total variou entre 91,0% e 96,0% para as diferentes fases, reforçando a percepção de alta validade de conteúdo. Recomendados na literatura para validação de conteúdo^(13,14)^, esses índices evidenciam que o material do curso foi bem aceito quanto à relevância, clareza e aplicabilidade. O achado é fundamental para a aceitação do curso por futuras edições/turmas, ao garantir que atende aos critérios de qualidade necessários para o ensino em contextos de saúde e enfermagem.

O empreendedorismo é fenômeno de ação que exige protagonismo do profissional de saúde. No entanto, existe certa desconfiança sobre a possibilidade de se ensinar as características empreendedoras, já que fatores pessoais e contextuais influenciam diretamente esse perfil. Elementos como ter pais empreendedores e possuir experiência prévia no mercado de trabalho, entre outros, podem influenciar significativamente o desenvolvimento de competências empreendedoras e dificultar a mensuração dos efeitos de cursos voltados a essa temática^(20)^. Essa observação se alinha com o comentário do Juiz 1, que apontou a insuficiência de docentes e profissionais com formação em empreendedorismo, os efeitos negativos dessa lacuna na formação de enfermeiros empreendedores e a necessidade de desenvolver tais competências nos profissionais atuantes.

Já na primeira rodada de avaliação, a concordância entre os juízes destaca ainda a eficácia da metodologia utilizada na elaboração do curso. Embora dispensada a segunda rodada de avaliação, as sugestões fornecidas pelos juízes foram consideradas para ajustes e aprimoramento de edições futuras. A ausência de modificações significativas na estrutura ou conteúdo, somada à concordância inicial, permitiu considerar o curso validado já na primeira rodada de avaliação. Isso atesta o impacto positivo do planejamento e desenvolvimento de sua estrutura considerando as lacunas e necessidades evidenciadas no território, conforme evidenciado pelo Juiz 9.

As sugestões e comentários nas diferentes fases de análise reforçam que o curso está voltado para estimular nos enfermeiros uma visão crítica da prática profissional. O conteúdo evidencia a necessidade de aprimoramento e qualificação em áreas que extrapolam a dimensão técnico assistencial, contemplando também aspectos que envolvem as nuances do cuidado. Isso inclui desde instrumentos para identificação e vínculo com a clientela até ferramentas para superar barreiras relacionadas à burocracia, gestão de negócios, finanças, precificação de serviços e captação de clientes^(6)^ - aspectos valorizados pelos participantes.

Em contrapartida, autores críticos da educação empreendedora no Brasil argumentam que ela pode ser utilizada para disseminar o empreendedorismo como um modelo normativo que induz no estudante o desejo de ter seu próprio negócio e de aceitar riscos, responsabilizando-o integralmente por sua formação, sucesso ou fracasso. Justificam que esse modelo pode representar uma pedagogia que reduz a expressão de formas alternativas de conceber a educação enquanto direito do cidadão e bem comum^(21)^. Outros defendem que o empreendedorismo pode tornar-se um aparelho ideológico voltado à reprodução sociometabólica do capital(22). No entanto, ressalta-se que a atual visão ampliada de empreendedorismo, entendida como ferramenta de educação para o desenvolvimento de competências, extrapola os limites da atuação e adentra no campo do propósito profissional, da geração de vínculos e da criação de valor para o cliente.

### Limitações do estudo

Este estudo apresenta algumas limitações inerentes ao método adotado. A primeira refere-se ao número reduzido de juízes participantes, o que pode comprometer a generalização dos resultados e impactar a robustez estatística da validação de conteúdo. Embora todos os itens tenham atendido aos critérios de concordância estabelecidos, a pequena amostra limita a confiabilidade dos achados. Além disso, ressalta-se a possibilidade de viés subjetivo nas avaliações dos juízes, ou seja, eles podem ter sido influenciados por suas experiências pessoais e formação profissional, gerando interpretações individuais dos itens do instrumento. Não obstante os achados indicarem a validade de estrutura e conteúdo do curso, sua qualidade pode ser continuamente aprimorada a cada nova edição por meio de abordagens complementares, como a inclusão de um maior número de juízes, feedback qualitativo mais detalhado e a avaliação do impacto na prática profissional.

Contribuições para a Área da Enfermagem, Saúde ou Política Pública

O “Curso de Introdução ao Empreendedorismo e Inovação em Enfermagem e Saúde” apresenta-se como um recurso validado para a prática educacional em enfermagem e saúde, respeita a necessidade didático-pedagógica de seu cenário de inserção e promove uma formação mais alinhada com as necessidades emergentes de inovação e gestão na saúde. Contribui para o currículo da enfermagem e demais profissionais que dele se beneficiem ampliando os cenários de inserção e atuação profissional. Serve como ferramenta para fortalecer as competências profissionais, permite uma prática mais autônoma e inovadora bem como capacita profissionais para enfrentar os desafios contemporâneos.

## CONSIDERAÇÕES FINAIS

O “Curso de Empreendedorismo e Inovação em Enfermagem e Saúde” demonstrou elevada validade de estrutura e conteúdo, evidenciando solidez teórica e metodológica, além de potencial para ser replicado e fomentar a formação empreendedora em enfermagem e saúde. Ele contribui para estabelecer uma nova visão profissional voltada à busca pela inovação, planejamento contínuo e agregação de valor profissional. Fortalece o acesso a dispositivos formativos para o empreendedorismo em enfermagem e saúde bem como amplia a visibilidade e as oportunidades profissionais.

## Data Availability

Os dados de pesquisa estão disponíveis em repositório: https://doi.org/10.17605/OSF.IO/PCSYB.
